# A fully computational and reasonable representation for karyotypes

**DOI:** 10.1093/bioinformatics/btz440

**Published:** 2019-06-22

**Authors:** Jennifer D Warrender, Anthony V Moorman, Phillip Lord

**Affiliations:** 1 School of Computing, Newcastle University, Newcastle-upon-Tyne NE4 5TG, UK; 2 Northern Institute for Cancer Research, Newcastle University, Newcastle-upon-Tyne NE1 7RU, UK

## Abstract

**Summary:**

The human karyotype has been used as a mechanism for describing and detecting gross abnormalities in the genome for many decades. It is used both for routine diagnostic purposes and for research to further our understanding of the causes of disease. Despite these important applications there has been no rigorous computational representation of the karyotype; rather an informal, string-based representation is used, making it hard to check, organize and search data of this form. In this article, we describe our use of OWL, the Ontology Web Language, to generate a fully computational representation of the karyotype; the development of this ontology represents a significant advance from the traditional bioinformatics use for tagging and navigation and has necessitated the development of a new ontology development environment called Tawny-OWL.

**Availability and implementation:**

The Karyotype Ontology and associated Tawny-OWL source code is available on GitHub at https://github.com/jaydchan/tawny-karyotype, under a LGPL License, Version 3.0.

## 1 Introduction

The genetic complement of organisms is carried on one or more chromosomes. These chromosomes have a characteristic organization and, in many cases, a characteristic cytogenetic appearance. The analysis of this appearance has been known to relate directly to the underlying genetics for many years and, in fact, before the mechanistic link between the two was well understood. It remains of vital diagnostic importance, as well as providing a key tool for a large research community.

Human karyotypes are represented using *the International System for Human Cytogenetic Nomenclature* (Shaffer *et al.*, 2012). In essence, this represents the karyotype as a structured string (which we call ISCN strings) which describes the chromosome complement of an individual human, cell or, even, a mix of cells in an individual cell line. This system is highly developed having evolved over many years, heavily used and richly expressive; however, ISCN strings do not have good computational properties. Unlike string representations, such as InCHI (Heller *et al.*, 2015), ISCN strings lack a formal interpretation or a concise computational representation. Likewise, their specification is informal, indeed, it has no electronic representation and is not searchable. This causes significant difficulties for both small- and large-scale use as well as manipulation of karyotypic information: it is not straight-forward, for example, to validate that an individual string fulfils the specification, nor to search a large number of karyotypes to find those that fulfil some criteria. However, the formal representation of the diseases/disorders caused by these ISCN strings has been previously modelled in vocabularies such as the National Cancer Institute Thesaurus (NCIT) (Hartel *et al.*, 2005) and Orphanet Rare Disease Ontology (ORDO) (Vasant *et al.*, 2014). While we cannot use these to reason over, ORDO could be a useful source of annotation for modelling Karyotypic diseases.

In this article, we describe a new representation for human karyotypes, the *Karyotype Ontology*. It has been defined using OWL, the Ontology Web Language, which means that it has a formal interpretation and specification. Unlike many traditional bio-ontologies, it makes extensive use of the expressivity of OWL, which means that karyotypes can be validated. With the use of a computational reasoner, it is possible to express queries enabling search against a large number of karyotypes. In addition, we describe the methodology that we have used to develop this ontology. To allow validation and searching, the ontology makes extensive use of complex, but repetitive expressions in OWL; for this reason, it has been built programmatically, using the Tawny-OWL library (Lord, 2013). This has also allowed us to develop a full suite of unit tests, test the scalability of the reasoning and to investigate the effect of different representations on this scalability.

## 2 What is an ISCN string

The human karyotype is normally represented using a string following the conventions defined in the International System for Human Cytogenetic Nomenclature (ISCN). First published around the 1960s, this system has been updated regularly subsequently. Although unnamed in the specification, here we call a string following these conventions an *ISCN string*.

**Fig. 1.**
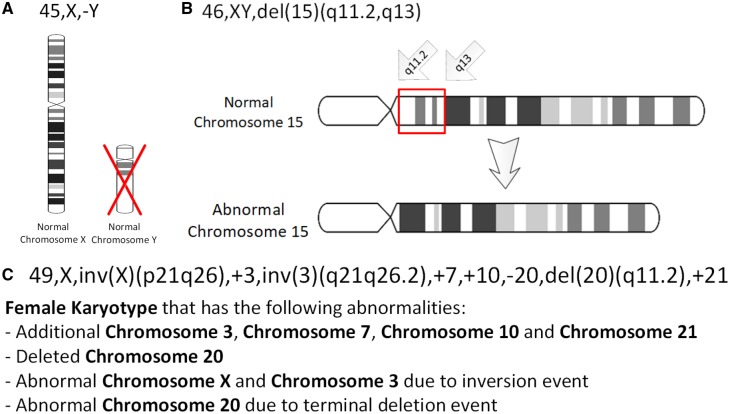
Three example ISCN strings that show an increase in complexity. Specifically, (**A**) A tumour karyotype in a male with loss of the Y chromosome, (**B**) Prader–Willi Syndrome i.e. deletion in the 15q11-q12 region and (**C**) an arbitrary karyotype that involves a variety of autosomal and allosomal abnormalities

Unsurprisingly, for a specification of this age the ISCN specification is informally defined. It does not define a computationally interpretable grammar nor is there a formal interpretation or underlying semantics. Similarly, the specification itself is not available in a computationally amenable or even an electronic format, meaning that is not even straight-forwardly searchable. As a result of this ISCN strings are difficult to parse, validate and query, especially for complicated ISCN strings (Fig. 1, for example, shows the representation of Prader–Willi syndrome).

There are a number of different approaches that could be taken to describe a karyotype: at heart, the karyotype is a description of the chromosomes and, underlying this, the genome of the organism in question. One potential way to represent the karyotype, therefore, would be to simply describe all the chromosomes and bands present. Unfortunately, with this approach, all the chromosomes and the bands must be described for every karyotype, which would be a fairly inefficient representation; additionally, a representation of this form would be hard to interpret for the user.

The ISCN therefore takes the approach of representing the karyotype as a series of changes, or events; we might say that a karyotype has lost a single chromosome 1, rather than enumerating all the chromosomes that it does have. There are a large number of changes that can happen to a chromosome, and these are described in Table 1. For any particular karyotype, these *changes* are notional and interpretative; they are not a description of the changes that definitely have happened but those that could have happened to produce the observed karyotype. For instance, a whole chromosome loss could involve the loss of the p-arm, and then the later loss of the q-arm. From this perspective, ISCN is rather like an edit distance as opposed to a description of history.

**Table 1.** List of events present in the ISCN which can be represented using the Karyotype Ontology

add	Addition
del	Deletion
der	Derivative Chromosome
dic	Dicentric Chromosome
dup	Duplication
fis	Fission
fra	Fragile Site
hsr	Homogeneously Staining Region
ins	Insertion
inv	Inversion
i	Isochromosome
mar	Marker Chromosome
neo	Neocentromere
qdp	Quadruplication
r	Ring Chromosome
tas	Telomeric Association
t	Translocation
trc	Tricentric Chromosome
trp	Triplication

## 3 Representing karyotypes

There are a large number of different technologies that could be used to represent a karyotype, mostly obviously a relational or XML based data model. Both of these would be capable of solving one immediate problem with ISCN strings—that is of the surface syntax. However, the underlying semantics are still complex and difficult to adequately represent. Therefore, we choose to use an ontological representation using the Ontology Web Language; this has a relatively rich semantics for representing categorical statements similar to those in the ISCN. We call this representation, the *Karyotype Ontology*.

As with ISCN, there are a number of different ways that we could represent the karyotype and some of the same issues are raised; for example, our initial experiments encouraged us to follow the lead of ISCN and use an event-based model. Therefore, all the forms of modification described in ISCN have been directly represented in the Karyotype Ontology.

As well as the type of modification, we wish to be able to describe the locations on the chromosome that the modifications affect; therefore, the Karyotype Ontology has a direct representation of all 23 chromosomes and further the 800+ bands that they contain; this means that it is not possible to specify a chromosome or chromosome band that does not exist.

We wished to be able to describe *regions* of chromosomes, enabling us to state, for example, that a deletion covered from 1p34 to 1p32. The Karyotype Ontology therefore explicitly describes the organization and layout of the chromosome bands.

We also have a set of non-functional requirements. Our purpose for building the Karyotype Ontology was to enable a searchable and computationally tractable description of a human karyotype designation. We wished the ontology to be as small and as simple as possible, to ensure that any reasoning will happen as quickly as possible.

## 4 Building the karyotype ontology

The human karyotype has 23 chromosomes and around 800 bands (at different resolutions). Representing this ontologically presents a practical barrier: most ontology tools are designed for a person to create most classes and, based on our requirements, the Karyotype Ontology would need to have over 800 classes. While many ontologies are larger than this, the Karyotype Ontology is highly repetitive, with most classes following a standard pattern; moreover, we wished to maintain the flexibility of changing the axiomatization of the Karyotype Ontology. All of this would have been challenging with existing tools.

For all of these reasons, the Karyotype Ontology was built using a new tool, Tawny-OWL, that was motivated by this use case. Specifically, Tawny-OWL is an ontology development environment implemented as a Domain Specific Language (DSL) in the programming language Clojure. This provides the ontology developer with a simple syntax (modelled on the OWL Manchester Syntax, Horridge and Patel-Schneider, 2012) with which to build their ontology, embedded in a complete programmatic language which provides: an evaluative shell, or REPL; functions for building patterns or other extensions; a unit test framework. In addition, the wider Clojure ecosystem provides development tools such as: IDEs or power editors with access to version control; code browsers; debuggers; build and deployment tools. In this article, we describe the ontology mostly using Tawny-OWL syntax, with one translation to Manchester syntax for comparison: full details of Tawny-OWL syntax are available in the manual (see http://homepages.cs.ncl.ac.uk/phillip.lord/take-wing/take_wing.html).

The Karyotype Ontology is therefore implemented using Tawny-OWL. The chromosomes and their banding patterns are written as literal data structures in code; these are then converted using patterns implemented in Clojure and Tawny-OWL into a set of ontological axioms using the OWL API (Horridge and Bechhofer, 2011). The key advantage of this approach is that it is possible to redesign the patterns which produce the ontological representation freely, and then update the entire ontology so that it is consistent against the redesign.

**Fig. 2.**
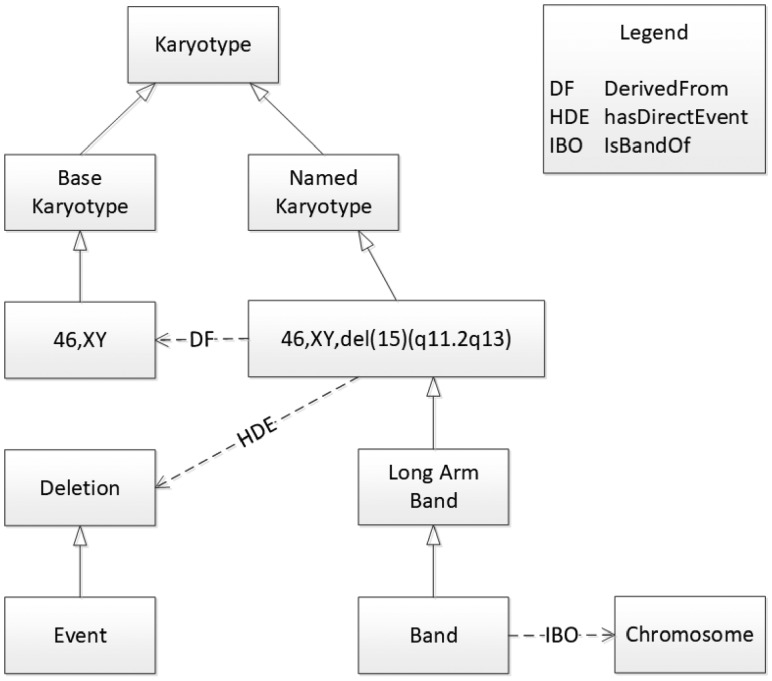
Some of the key entities in the Karyotype Ontology as they apply to describing the Prader-Willi karyotype

Next, we describe the overall organization of classes and properties in the Karyotype Ontology; we illustrate this in Figure 2, showing how they are applied to describe the Prader-Willi karyotype shown earlier.

The human karyotype is modelled (perhaps obviously) as a partonomy, with some inheritance. We actually use properties called ‘isBandOf’; as we do not use an upper ontology, these are not related to a property with the name ‘part-of’. So, for example,

HumanChromosome1Bandp35 is a HumanChromosome1p which is a band of HumanChromosome1 which is, itself a HumanAutosome, which is a HumanChromosome. The ‘open world’ semantics of OWL means that we also need to make explicit the disjointness between bands and chromosomes. This is done at several different levels to minimize the total number of disjoint axioms that need to be made: for example, HumanChromosome1 is disjoint with HumanChromosome2 but not HumanChromosomeX, as the sex chromosomes and autosomes are already disjoint. Likewise, bands are only directly disjoint from bands on the same chromosome arm.

**Fig. 3.**
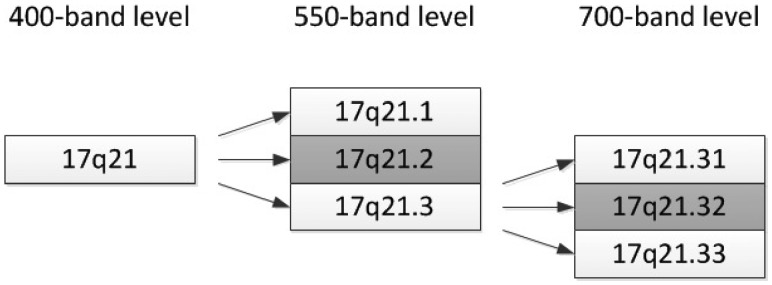
Visualizing the higher resolution sub-bands of human chromosome band 17q21

We also explicitly model resolution. As Clojure symbols cannot start with numbers all resolutions and karyotypes start with the **r** and **k** character, respectively. The human karyotype has more visible bands at higher resolutions, and these need to be explicitly described (see Fig. 3). We achieve this with a class and object property. Similar to prefixes in OWL we use Clojure namespaces to refer to entities that are defined in other namespaces. As with *named* karyotypes, we provide a number of defined classes.

(defclass r300-band)

(defoproperty seenAtResolution

 :domain h/HumanChromosomeBand

 :range Resolution)

(defclass is-300-band

 :equivalent

 (owl-and

  h/HumanChromosomeBand

  (owl-some seenAtResolution r300-band)))

This is syntactically very similar to the equivalent statements in Manchester Syntax which we show here for reference:

Class: res: r300-band

  SubClassOf:

    res: Resolution

ObjectProperty: res: seenAtResolution

  Domain:

    hum: HumanChromosomeBand

  Range:

    res: Resolution

Class: res: is-300-band

  EquivalentTo:

    hum: HumanChromosomeBand

    and (res: seenAtResolution

        some res: r300-band)

As well as the human chromosome structure, we model a large number of ‘features’ such as fragile sites, neocentromeres and ring chromosomes, all of which are defined in the ISCN. These are all associated with functions which can be used to define restrictions describing these features of chromosomes. For example, the statements in this listing define an ontology class, FragileSite and the pattern function fragilesite which uses this class. In Clojure, we define functions using the reserved keyword defn and parameters are declared within the square brackets. fragilesite itself uses a second pattern which, in this case, expands to an OWL ‘some’ restriction.

(defclass FragileSite)

(defn fragilesite

 [n band]

 (direct-feature

 n

 (owl-and FragileSite

 (owl-some e/hasBreakPoint band))))

The main purpose of this use of a function is simply to provide additional syntax, which reduces the amount of typing but, as with the use of patterns defining the chromosome structure, it also provides a degree of abstraction, meaning the pattern could be updated.

In a similar vein, we also model the ISCN events, describing changes that can happen to a chromosome. As with features, these are defined in pairs defining an OWL class and a pattern. For example, an addition of a band is implemented as follows:

(defclass Addition)

(defn addition-band

 [band]

 (owl-and

  Addition

  (owl-some hasBreakPoint band)))

For events to be meaningful, we have to provide some start point to which these events can happen. We define these simply such that their composition is not explicitly either in terms of their chromosomes or chromosome bands. For example, the diploid karyotypes are defined as follows:

(defclass k46_XN

 :super BaseKaryotype)

(as-disjoint-subclasses

 k46_XN

 (defclass k46_XX)

 (defclass k46_XY))

In order to create a Clojure symbol that was also legal in Manchester Syntax, the commas have been replaced with underscores. We also define haploid, triploid and tetraploid karyotypes, and a large number of *named* karyotypes associated either with biological conditions (e.g. Male or Female karyotypes) or specific syndromes (e.g. trisomy 21, or Down Syndrome). These latter are taken from examples given in the ISCN—we have not encoded all the karyotypes simply because there are a very large number, but have sampled across most sections of the book. A set of karyotype kinds are also stated as *defined* classes, that can be used in conjunction with a computational reasoner as queries: for example, a structural or numerically abnormal karyotype (defined below).

(defclass NumericalAbnormalKaryotype

 :equivalent

 (owl-or

  (e/event

  nil

  (e/addition-chromosome

   h/HumanChromosome))

 (e/event

  nil

  (e/deletion-chromosome

   h/HumanChromosome))))

Taken together, this enables us to express karyotypes of arbitrary complexity. For example, consider the representation of the ISCN string 45, X,-Y that we saw in Figure 1; this karyotype can be seen as arising from a single event happening to a base karyotype.

(defclass k45_X_-Y

 :super

 (owl-some b/derivedFrom b/k46_XY)

 (e/deletion 1 h/HumanChromosomeY)

This representation is clear and unambiguous and can be reasoned over; for example, it can be retrieved as a diploid, male karyotype with loss of the Y-chromosome.

In the next section, we describe our testing strategy which ensures that this reasoning works as expected.

## 5 Testing the karyotype ontology

The Karyotype Ontology is a relatively complex ontology, containing some rich axiomatization. This reason alone would make it sensible to test, to ensure that the ontology has been built correctly. There is, however, a more compelling reason in this case; our intention is that with the Karyotype Ontology it should be possible to query over a large number of karyotypes defined using this ontology, for those which fulfil a given set of criteria using computational reasoning. For this, we need to ensure that the ontology reasons correctly.

This has been achieved through combination of the Clojure unit test framework, and Tawny-OWL’s interface to the computational reasoner HermiT. Tests are defined into two halves; first, we have picked a large number of the classes defined in the Karyotype Ontology for testing.

For example, the following statements assert that Down syndrome is a diploid karyotype with an autosomal gain, and that 46, XY is NOT female. We do this using the **is** macro, found in Clojure’s testing framework, to make these assertions.

(is

 (r/isuperclass?

  n/DownSyndrome

  n/DiploidKaryotype))

(is

 (r/isuperclass?

  n/DownSyndrome

  n/NumericalAbnormalKaryotypeAutosomalGain))

(is

(not

  (r/isuperclass?

   b/k46_XY

   n/FemaleKaryotype)))

In addition, we have classified a large number of different example karyotypes from ISCN against 17 different defined classes (diploid, female, fission and so on). Writing all these tests by hand would have been long-winded so, instead, they are encoded in a spreadsheet, following a document-centric approach (Blfgeh *et al.*, 2017) and mirroring existing template-based ontology tools such as Populous (Jupp *et al.*, 2010). This spreadsheet is directly parsed as part of the Karyotype Ontology test cycle. In addition, we also use this to specify whether the karyotype is parsable from the ISCN string, an additional functionality of the Karyotype Ontology code base.

## 6 Optimizing the karyotype ontology

The primary intention of the Karyotype Ontology is to provide an implementation which is capable of machine interpretation and can be reasoned over. This is not true for many ontologies which aim to provide a controlled vocabulary or to aid navigation (Stevens and Lord, 2009). One key concern, therefore, during the construction of the ontology was that it is efficient and can be reasoned over. This is a concern for any ontology written in OWL-DL such as the Karyotype Ontology. We demonstrate some of the considerations necessary when describing ordering of the chromosome bands.

The human karyotype contains bands which are necessarily ordered, and this order is necessary to understand the impact of a number of aspects of a given karyotype. For instance, a deletion from 1p31 to 1p21 will also impact on all the sequences in band 1p22 (and 1p31 and 1p21), since this band is between the two breakpoints, but only these since only 1p22 is between these two.

Ontologies are generally not good at representing order, as their underlying data structures are, similarly, not ordered. When considered as a tree or directed acyclic graph, leaf nodes are not ordered with respect to each other; likewise, the formal logical representation of OWL treats most elements as a (disordered) set. Despite not having formal support for ordering, there are a number of ways in which it can be achieved; we consider four possibilities here.

- **No Order:** Order is not represented.
- **Enumeration:** All the affected bands are explicitly stated in the model, using a Tawny-OWL pattern to expand from a start-point to an end-point.
- **Sequence Pattern:** This ontology design pattern (Drummond *et al.*, 2006) describes a sequence, using a ‘next’ to and ‘rest’ relationship. ‘next’ describes links to an item, and ‘rest’ another list.
- **Data Properties:** Bands are given an integer value, using OWLs datatype support to enable comparison.

These have different characteristics in terms of expressivity, ease of use and so forth. The most important characteristic, though, which is query/reasoning performance is almost impossible to predict *a priori* and may, anyway, vary between different extant reasoners or a single reasoner over time.

**Fig. 4.**
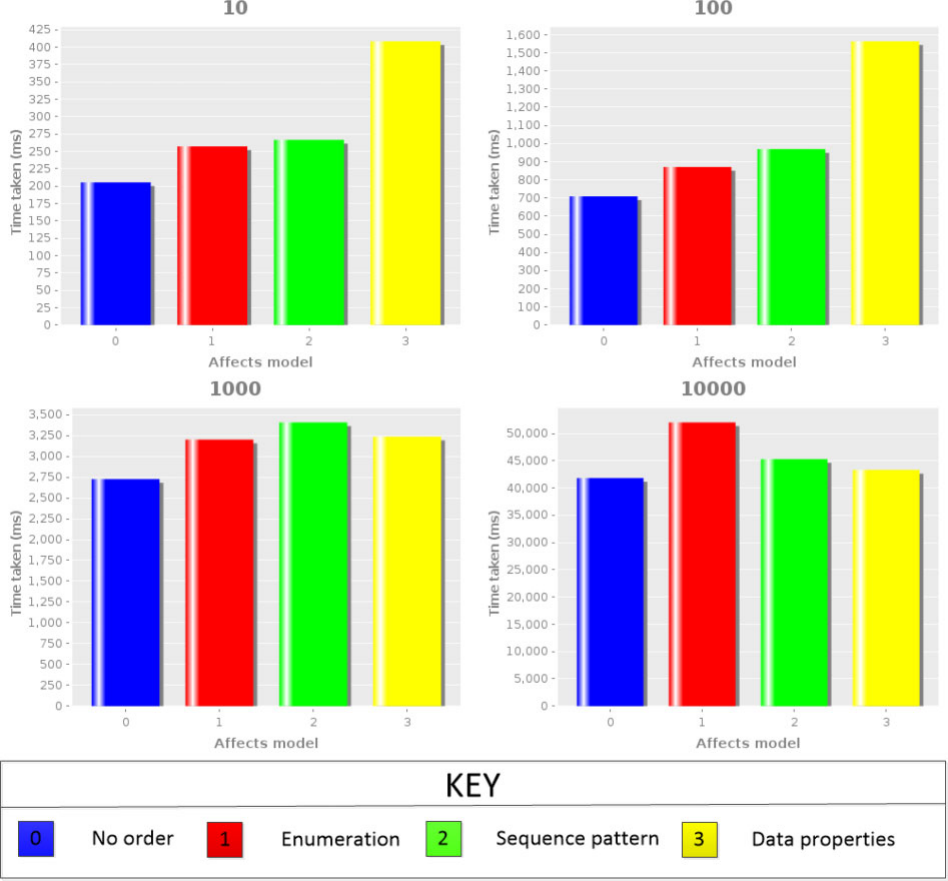
Bar charts showing the mean reasoning times for each affects implementation from 101 to 104 number of karyotypes. As shown in the key, the first bar represents reasoning times for the original representation (0), while the following three bars represent the reasoning times for enumeration (1), sequence patterns (2)and data properties (3) implementation, respectively

One interesting consequence of the pattern-driven approach used to develop the Karyotype Ontology is that it is possible to change the patterns and then regenerate the ontology. In short, we can test these four different possibilities. This was achieved by generating large numbers of random karyotypes, and then testing (see https://github.com/jaydchan/tawny-karyotype-scaling). Results are shown in Figure 4.

From these results, we can see that the reasoning performance of the Karyotype Ontology is entirely usable, at under a minute for 10 000 karyotypes, that further adding ordering information to the Karyotype Ontology does not add excessive time to the overall reasoning, and that all three different mechanisms for representation of order have acceptable performance. However, we note that the different representations scale differently and that the choice is dependent on the number of karyotypes being searched. While, we note that while we supported several axiomatizations to enable performance testing during development, it could also be used during deployment, as a mechanism for scaling a karyotype database.

## 7 Discussion

In this article, we have described the development of the Karyotype Ontology. This ontology provides the ability to describe the human karyotype. Its representation closely follows the ISCN standard and is strongly based upon it. We have followed this approach for a variety of reasons. It is obviously most familiar to biologists and clinicians who deal with this data. Most importantly, ISCN has been built up and refined over many years and contains a lot of knowledge, and is likely to be a good representation.

**Fig. 5.**
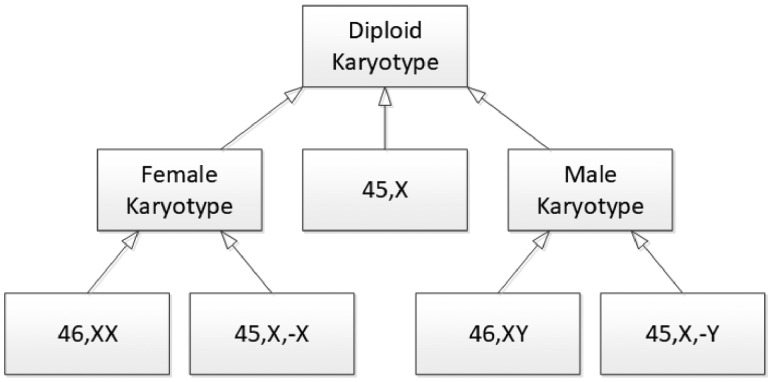
The inferred class hierarchy for classes describing sex. None of the links in this diagram are explicitly stated

We have considered the possibility of building the Karyotype Ontology purely as a partonomy; that is a representation that describes the human karyotype on the basis of the chromosomes and chromosome bands that it has. Initially, we decided against this representation on the grounds of performance. However, there are subtler reasons why this is not appropriate. For example, the consider the karyotype 45, XO which manifests as Turners Syndrome; that it is has a single X-chromosome. Partonomically, this is identical to the karyotype 45, X,-Y which also has a single X-chromosome. However, the latter karyotype is of a cell-line that has lost a Y-chromosome, while the former is a congenital ‘loss’. We can only distinguish these two on the basis of their history. This example also illustrates a surprising use of reasoning within the ontology (see Fig. 5). Intuitively, you would assume that a male would be defined as any karyotype with a Y-chromosome; however, 45, X,-Y should be considered to be male even though it has no Y; the Karyotype Ontology, therefore, defines a male karyotype as one *derived from* 46, XY. This definition means 45, X,-Y is male; Turners syndrome, although phenotypically female, can be reasoned neither to be male nor female since the Karyotype Ontology defines to be derived from 46, XN—we do not know which kind of chromosome it has lost; it would, of course, be possible to assert this knowledge, once it is clear what answer the community would expect.

The Karyotype Ontology is also not an ontology in the realist mould (Lord and Stevens, 2010). We neither represent the partonomy as it exists, nor claim to represent the actual, historical changes that have been made during the course of the development of the cell or organism. In addition, we have followed the pragmatic approach of making it as simple as possible: we have eschewed making distinctions that we do not need to fulfil our computational objective; we have not used an upper ontology; and, we have not cross-linked to other bio-ontologies. These are not omissions; they have been avoided as they do not fulfil the direct use case; if we wished to add these, they would be added to a secondary ontology which could import the Karyotype Ontology. The Karyotype Ontology is a computational representation of a specification to enable searching of karyotypes, and fulfils this function. In this sense, the Karyotype Ontology is quite a different form of ontology from many others seen in bio-medical ontologies.

In addition to this, we have adopted a different development style from many others. This ontology provided us a use case for the Tawny-OWL library, which enables fully programmatic ontology development. This has allowed us to adopt most of the industry-standard practices and tools from software engineering including functional abstraction, repeatable builds and unit tests, as well as tools such as an IDEs, versioning and continuous integration. This also gives us the ability to change wholescale our axiomatization by changing our patterns. We have used this ability to test the performance impact of different axiomatizations; to our knowledge this is the first time, this form of large-scale performance testing has been used on a complete ontology.

There are, of course, some limitations to the Karyotype Ontology. We believe that the semantics of the representation are a considerable improvement over the existing ISCN strings in terms of computational precision and formality and, further, the syntax is defined, parsable and works well with existing tooling; however, the current representation is too verbose and difficult to write to directly to take the place of these strings. We have developed a parser that will convert some ISCN strings into the Karyotype Ontology, but given the informal definition of ISCN this is obviously difficult and heuristic; in future, we hope to develop a formal string representation which can compile to the OWL representation, similar to the way that InCHI strings can be converted to a chemical structure.

We also note that the Karyotype Ontology is currently specifically a representation of the human karyotype; it would require modification or extension for use with other organisms. Given the difference in representation between different communities, it is likely that only a few high-level terms would be sharable. However, the generalized methodology that we have developed would be applicable. In this sense, as well as providing the first highly computational representation of the human karyotype, we have also introduced a new methodology for ontology development, recasting ontologies from tools for tagging records to a tool for modelling a complex area of biology accurately, precisely and searchable. We expect that there are many further areas of biology where this will prove to be useful.

## Funding

This work was supported by the Engineering and Physical Sciences Research Council [EP/P50564X/1].

*Conflict of Interest*: none declared.
